# Assessing harmonized intelligence measures in a multinational study

**DOI:** 10.1017/gmh.2024.22

**Published:** 2024-02-23

**Authors:** Mariah DeSerisy, Melanie M. Wall, Terry E. Goldberg, Marcelo C. Batistuzzo, Katherine Keyes, Niels T. de Joode, Christine Lochner, Clara Marincowitz, Madhuri Narayan, Nitin Anand, Amy M. Rapp, Dan J. Stein, H. Blair Simpson, Amy E. Margolis

**Affiliations:** 1 Columbia University Medical Center, Mailman School of Public Health, Columbia University, New York, NY, USA; 2 Columbia University Irving Medical Center, Columbia University, New York, NY, USA; 3 Department of Psychiatry, The New York State Psychiatric Institute, New York, NY, USA; 4 Department of Psychiatry, Faculdade de Medicina, Universidade de São Paulo, São Paulo, Brazil; 5 Department of Methods and Techniques in Psychology, Pontifical Catholic University, São Paulo, Brazil; 6 Department of Psychiatry, Amsterdam UMC, Vrije Universiteit Amsterdam, Amsterdam, Netherlands; 7 Department of Anatomy and Neuroscience, Amsterdam UMC, Amsterdam Neuroscience, Vrije Universiteit Amsterdam, Amsterdam, Netherlands; 8 SAMRC Unit on Risk & Resilience in Mental Disorders, Department of Psychiatry, Stellenbosch University, Stellenbosch, South Africa; 9 Department of Clinical Psychology, National Institute of Mental Health & Neuro Sciences (NIMHANS), Institute of National Importance (INI), Bangalore, India; 10 SAMRC Unit on Risk & Resilience in Mental Disorders, Department of Psychiatry and Neuroscience Institute, University of Cape Town, Cape Town, South Africa

**Keywords:** socioeconomic status, education, healthy participants, full-scale intelligence quotient

## Abstract

Studies examining the neurocognitive and circuit-based etiology of psychiatric illness are moving toward inclusive, global designs. A potential confounding effect of these associations is general intelligence; however, an internationally validated, harmonized intelligence quotient (IQ) measure is not available. We describe the procedures used to measure IQ across a five-site, multinational study and demonstrate the harmonized measure’s cross-site validity. Culturally appropriate intelligence measures were selected: four short-form Wechsler intelligence tests (Brazil, Netherlands, South Africa, United States) and the Binet Kamat (India). Analyses included IQ scores from 255 healthy participants (age 18–50; 42% male). Regression analyses tested between-site differences in IQ scores, as well as expected associations with sociodemographic factors (sex, socioeconomic status, education) to assess validity. Harmonization (e.g., a priori selection of tests) yielded the compatibility of IQ measures. Higher IQ was associated with higher socioeconomic status, suggesting good convergent validity. No association was found between sex and IQ at any site, suggesting good discriminant validity. Associations between higher IQ and higher years of education were found at all sites except the United States. Harmonized IQ scores provide a measure of IQ with evidence of good validity that can be used in neurocognitive and circuit-based studies to control for intelligence across global sites.

## Impact statement

As mental health research shifts toward more inclusive, global studies, there is an increasing need for a harmonized measure of intelligence for use in multinational studies in order to address the potentially confounding effects of intelligence on mental health outcomes. To date, no work has examined the convergent and divergent validity of harmonized intelligence scores across multiple study sites located in different countries. We demonstrated that the full-scale intelligence quotient (IQ) measure harmonized across five multinational sites correlated with socioeconomic status and educational attainment indicating convergent validity and showed that it did not correlate with sex indicating discriminant validity. Site-specific effects were observed and are discussed in the context of their implications for future analyses with combined data across these global sites. The confounding effect of individual differences in intelligence among individuals with neuropsychiatric disorders presents unique challenges for global investigations of mental health across different countries. Our data suggest that this can be mitigated by incorporating a prospectively harmonized valid measure of IQ into analyses to adjust for this confounding, providing preliminary support for using such an approach in future multinational studies.

## Introduction

Neuropsychiatric disorders account for as much as 10% of the disease burden worldwide (Santomauro et al., 2021); however, access to mental health care and research to support such care remains scarce (World Health Organization, Mental Health Determinants and Populations Team, 2001). Studies examining neurocognitive functioning in and neural circuitry of psychiatric illnesses are moving toward more inclusive and global designs. Such work raises the need to address challenges inherent in measuring neurocognitive abilities in different countries that may vary in terms of resources or language, factors known to be associated with performance on cognitive tests.

Intelligence testing is a commonly used tool in research to address individual differences in cognitive capacities across participants by measuring the ability to use information or abstract reasoning to answer questions, make predictions and learn from experience across a number of domains (Deary, 2012; Russell, 2020). Individual differences in intelligence are important to include in studies designed to measure cognitive problems associated with psychiatric diagnoses because intelligence is associated with psychiatric symptoms and with performance on cognitive tests (Abramovitch et al., 2018; Ruiz et al., 2020; Thompson et al., 2020a). The potentially confounding effect of individual differences in intelligence on cognitive performance presents unique challenges for global investigations of mental health across different countries especially given that there is no existing best practice for how to measure intelligence across different countries.

Intelligence quotients (IQs) are thought to measure global *g*, the theorized common factor representing human intelligence (Spearman, 1904). Global *g*, or *g*-factor, has been argued to represent a universal human phenomenon (Warne and Burningham, 2019; Russell, 2020); however, the way *g* manifests is likely to be context specific (i.e., skills useful in an urban context might be different in a rural context) (Warne and Burningham, 2019; Russell, 2020). As such, it is essential to interpret results from intelligence testing within the context of a specific country, region or study site in global studies of mental health.

Although global collaborations examining cognitive outcomes are increasing, the method for handling measures of intelligence has varied widely and has not focused on validity of the measure across sites. The majority of work that has incorporated IQ scores across multiple sites have leveraged a full-scale IQ score regardless of the assessment tool used (e.g., van Bakel et al., 2014; Sentenac et al., 2021) or in big data sets, aggregation is done restricting only to sites that have the same IQ measures (Bedford et al., 2020). Rarely have studies attempted to combine sites with different measures of intelligence from across continents (Mortillo and Mulle, 2021; Wallert et al., 2021). For example, Mortillo and Mulle (2021) combined data as well as types of tests across countries by comparing country-specific norm-referenced standard scores and also dichotomizing participants into groups based on intellectual disability status. In contrast, Wallert et al. (2021) utilized principal components analysis to extract a g-factor from multiple cognitive tests by combining data from participants in North America and Sweden. None of the studies provided any demonstration of the validity of the intelligence measure across sites. We address this gap by proposing to use prospectively harmonized compatible measures with country-specific norms and then to demonstrate that this harmonized measure shows convergent and discriminant validity across sites. Of note, data harmonization is a tool that can be used to maintain the integrity of context-specific data, such as IQ, while also pooling across contexts to facilitate large-scale global collaborations. Harmonization can be prospective, via careful selection of culturally relevant, reliable and valid measures occurring after data collection has started and leveraging statistical approaches to ensure data compatibility (Griffith et al., 2013, 2016).

From psychometrics, the validity of a measure is determined via its consistent associations with variables theoretically predicted to be related to it in specific ways, that is, convergent and discriminant validity (Campbell and Fiske, 1959). Intelligence is both heritable and malleable (Sauce and Matzel, 2018), with strong bidirectional associations with sociodemographic factors, including socioeconomic status (SES, Strenze, 2007) and education (Ritchie and Tucker-Drob, 2018; Lövdén et al., 2020; Feinkohl et al., 2021). Of note, SES and education have each been shown to differentially associate with verbal (Matarazzo and Herman, 1984; Bornstein et al., 1987; Shuttleworth-Edwards et al., 2004; Walker et al., 2009; Chapman et al., 2014) and perceptual abilities (Matarazzo and Herman, 1984; Bornstein et al., 1987; Shuttleworth-Edwards et al., 2004; Mani et al., 2013; Piccolo et al., 2016). In contrast, other demographic factors, such as sex, are less correlated with intellectual abilities. Sex differences in FSIQ have not been consistently found (e.g., Colom et al., 2002; Daseking et al., 2017; Halpern and Wai, 2019). However, there is evidence to suggest there may be sex-specific differences in performance on individual subtests or across specific domains (e.g., Irwing, 2012; Pezzuti et al., 2020). In sum, when attempting to confirm the validity of a cross-national IQ measure, we would expect to find positive correlations between the IQ measure, SES and education (convergent validity), but to find minimal or no associations between the IQ measure and sex (discriminant validity).

This manuscript reports on the prospective harmonization process used to select culturally appropriate IQ measures across sites from five countries collected as part of a study examining cognitive and neurobiological correlates of obsessive–compulsive disorder (OCD; Simpson et al., 2020) compared to healthy participants. We leverage the harmonized intelligence measure obtained from healthy participants to examine the measure’s convergent and discriminant validity across sites in comparison to sociodemographic factors.

## Methods

### Participants

The parent study recruited and evaluated a large and diverse sample of medication-free adults with OCD and matched healthy participants across five academic medical sites located in Brazil, India, the Netherlands, South Africa and the United States. A full description of the parent study protocol can be found elsewhere (Simpson et al., 2020). Given our focus on assessing the validity of the IQ measure across sites, we included only healthy control participants. Subjects with OCD may exhibit systematic differences in IQ (Abramovitch et al., 2018) that might be associated with the validity assessment. A total of 256 healthy participants (*n* = 255 with completed intelligence measure) were recruited across all five sites and selected to match the OCD sample in distribution on age, sex and educational level (within sites but not necessarily between sites). Healthy participants were aged 18–50 years and were not eligible to participate if they had a first-degree relative with OCD or tic disorder, current or past use of psychotropic medications or current or lifetime psychiatric disorder other than major depressive disorder or anxiety disorders (if not in past year). Importantly, healthy participants were also not eligible if they had an FSIQ score below 80.

### Prospectively chosen intelligence assessments

Intelligence tests were chosen in consultation with local experts to determine the most context-valid and appropriate test for use at each site, keeping in mind the need for compatibility across sites (Table 1). Thus, intelligence testing was performed using different instruments depending on the site location, local population characteristics and local dominant language. When available, preference was given to short forms of the Wechsler tests to minimize participant burden, reduce cross-site heterogeneity and maximize harmonization opportunities. Of note, discrete ability scores (Perceptual Reasoning Index [PIQ] and Verbal Comprehension Index [VIQ]) were derived whenever possible, as described below.

**Table 1.** Prospectively chosen intelligence measures across sites

Site	Intelligence measure
Brazil	Brazilian version of the Wechsler Abbreviated Scale of Intelligence, First Edition (WASI-I)
India	Binet Kamat Test of Intelligence
Netherlands	Four subscales from the Netherlands version of the Wechsler Adult Intelligence Scale, Fourth Edition (WAIS-IV-NL) selected to match the WASI subscales
South Africa	English version of the Wechsler Abbreviated Scale of Intelligence, Second Edition (WASI-II)
United States	English version of the Wechsler Abbreviated Scale of Intelligence, Second Edition (WASI-II)

#### Brazil

The Brazilian site (located in San Paolo) utilized the Brazilian version of the Wechsler Abbreviated Scale of Intelligence, First Edition (WASI-I; Wechsler, 1999; Trentini et al., 2014) administered in Brazilian Portuguese by bachelor’s level psychologist evaluators trained by a post-doctoral level psychologist. The WASI-I consists of Block Design, Matrix Reasoning, Vocabulary and Similarities subtests and derives an examinee’s PIQ, VIQ and FSIQ. Evaluators were trained to reliability and supervised by a doctoral level clinician with >10 years of expertise in neuropsychological assessment. All protocols were scored by the same professional, the supervisor, to ensure ongoing reliability. Tests were scored using publisher norms developed with Brazilian populations (Trentini et al., 2014).

#### India

The India site (located in Bangalore) utilized the Binet Kamat Test of Intelligence (Kamat, 1968) administered in English or Kannada by bilingual evaluators depending on the preference of the participant and based on their language proficiency. Notably, an Indian version of Wechsler tests is not available; therefore, the Binet Kamat was selected as an intelligence test with available local norms. The intelligence measure was administered by master’s level and doctoral-level student clinical psychology evaluators. The Binet Kamat Test includes both verbal and nonverbal items but does not consist of specific subtests or derive subtest scores. Instead, the Binet Kamat derives only an FSIQ score. Evaluators were trained to reliability by doctoral-level clinicians with expertise in neuropsychological assessment and supervised by a doctoral-level clinician. Every fifth test protocol was double-scored by the test administrator and a doctoral-level clinician to ensure ongoing reliability. Tests were scored using norms developed with Indian populations (Kamat, 1968). Despite their age, recent evidence suggests that these norms are still valid among Indian participants (Roopesh, 2020).

#### Netherlands

The Netherlands site (located in Amsterdam) utilized four selected subscales from the Netherlands version of the Wechsler Adult Intelligence Scale, Fourth Edition (Wechsler, 2009) administered in Dutch. Completed subtests included Block Design, Matrix Reasoning, Vocabulary and Similarities to match other sites and derive an examinee’s PIQ, VIQ and FSIQ. Evaluations were completed by doctoral students, master’s students and a bachelor’s level research assistant via iPads. Evaluators were trained to reliability by a doctoral-level clinician with expertise in neuropsychological assessment and supervised by a doctoral-level clinician. Every fifth test protocol was reviewed, and the Vocabulary and Similarities were double-scored by the test administrator and a doctoral-level supervisor to ensure ongoing reliability. Matrix Reasoning and Block Design subsets were automatically generated based on participants’ iPad responses. Tests were scored using publisher norms developed with Dutch and Flemish populations (Wechsler, 2009).

#### South Africa

The South Africa site (located in Cape Town) utilized the English version of the WASI, Second Edition (WASI-II; Wechsler, 2011) administered by bilingual master’s and doctoral-level evaluators. Participants completed the test in either English or Afrikaans, depending on the preference of the participant and based on their language proficiency and the language in which they completed the majority of their education. Of note, an Afrikaans version of Wechsler tests is not available; however, the majority of South Africans in the catchment population were bilingual. Specifically, the majority of participants reported their first language as Afrikaans but performing most educational and occupational duties in English. Test items and directions from the English version of the WASI-II were translated to Afrikaans by bilingual study team members to produce a standardized Afrikaans assessment; of note, test items were directly translated from English to Afrikaans, which may or may not preserve the intended item difficulty. When requested (*n* = 18), the translated assessment was presented. The WASI-II consists of Block Design, Matrix Reasoning, Vocabulary and Similarities subtests and derives an examinee’s PIQ, VIQ and FSIQ. Evaluators were trained to reliability and supervised by a doctoral-level clinician with expertise in neuropsychological assessment. Every fifth test protocol was reviewed and the Vocabulary and Similarities were double-scored by the test administrator and the doctoral-level supervisor to ensure ongoing reliability. Tests were scored using U.S. publisher norms, as South African norms are not available for the WASI-II. Notably, an alternative test instrument with local norms has not been developed. Cross-site reliability was assessed through monthly meetings with the U.S. site (also utilizing the WASI-II) in which a team of six raters independently rated a test protocol and scores were determined by consensus.

#### United States

The U.S. site (located in New York City) utilized the WASI-II (Wechsler, 2011) administered in English. PIQ, VIQ and FSIQ were derived. Evaluators consisted of bachelor’s level research assistants trained to reliability in the administration of the WASI-II. Evaluators were trained by doctoral-level clinicians with expertise in neuropsychological assessment and supervised by a doctoral-level clinician. Every fifth test protocol in entirety was double-scored by the test administrator and a doctoral-level supervisor to ensure ongoing reliability. Tests were scored using publisher norms developed with U.S. populations. As described above, cross-site reliability was assessed through monthly structured meetings with the South Africa site.

### Sociodemographic factors for assessing convergent and discriminant validity

*Educational attainment*, or years of education, is known to be associated with IQ scores and interact with SES (Ritchie and Tucker-Drob, 2018). Further, it has been used to approximate SES because it can be obtained for all participants, in contrast to other measures such as occupation or income that are associated with family structure (i.e., stay-at-home parents) and retirement age, and is typically robust to late-life health impairments (Liberatos et al., 1988; Elo and Preston, 1996). In this study, years of education refers to the number of completed (i.e., passed) years of schooling, beginning with the first grade, and was prospectively determined to be a valid harmonized measure across the five countries. Additionally, this method of measuring educational attainment has been used in previous multinational studies (Thompson et al., 2020b).

*The WAMI Index* (Psaki et al., 2014) measures access to resources and living conditions that differ between developing and developed countries, such as access to improved water/sanitation, assets (e.g., housing resources), maternal education and income. The WAMI provides a summary index score as well as section scores examining 1) water/sanitation; 2) assets (e.g., possessions, number of rooms in the family home); 3) maternal educational attainment and 4) household income in local currency. This measure was prospectively chosen as it has been shown to validly measure SES across different countries (Psaki et al., 2014; Pradhan et al., 2018).

*Sex* was determined by the participant’s self-report.

### Data analytic plan

Descriptive summaries (means, standard deviations) and one-way analysis of variance or chi-squared (*χ* ^2^) tests were used to assess differences between sites in participant sociodemographic characteristics and IQ measures: FSIQ, VIQ and PIQ. Scheffé post hoc tests were used to test pairwise mean differences. To assess the construct validity (Terwee et al., 2007; Mokkink et al., 2010), we hypothesized that FSIQ, VIQ and PIQ will all correlate positively with years of education and WAMI (convergent validity) and will not correlate with sex (discriminant validity). Further, we hypothesized these associations to be found across sites and within each site. To test these validity hypotheses, we used general linear models (GLMs) for each IQ measure as the outcome predicted by site, sex, education and WAMI and included an interaction of site with each of the four sociodemographic measures to test the similarity of associations across sites. Given the known associations between age and IQ (i.e., declines in processing speed and fluid reasoning beginning in early adulthood and becoming impairing in elderly individuals age > 75; Miller et al., 2009; Baxendale, 2011; Singh-Manoux et al., 2012; Kremen et al., 2014), we include age and age by site interactions in our models to control for potential confounding by age. We note that IQ test norms adjust for age (Wechsler, 2009), but we include age in our models to account for differences in ages across sites. The India site was excluded from the analyses of VIQ and PIQ as these subtests were not available. Effect sizes were determined using partial eta-squared (*η* ^2^). Common rules of thumb for qualifying the size of partial *η* ^2^ are that 0.01 is small, 0.06 is medium and 0.14 is large (Richardson, 2011). Analyses were performed using IBM SPSS Statistics version 28 (IBM Corp., Armonk, NY, United States) and alpha was set at *p* < 0.05 (two-tailed) for all analyses.

Many participants across the global sites were multilingual. Sensitivity analyses evaluated the effects of language proficiency as well as task and task administration on primary results. Language proficiency was determined by asking participants’ their preferred language and determining if that language matched the administration language. This dichotomous variable was then included in the GLMs described above and tested. Because the India site used the Binet Kamat and because task administration was nonstandard in the South Africa site, the primary analysis was conducted without including data from the South Africa or India sites.

## Results

### Participants

Similar numbers of healthy participants were recruited at all five sites (range, *n* = 50–53), with average age across sites ranging from 27.7 to 32.7 and the gender distribution from 35% to 54% male (Table 2). Supplementary Table S1 describes participants’ ethno-racial backgrounds in detail. Across the sites, years of education were higher than the general population of the world (8.0–8.7 years; “Average years of schooling”, n.d.; Barro and Lee, 2013) and of each country (“Average years of schooling”, n.d.), with the highest level in Brazil (17 years, country average = 8 years; “Average years of schooling”, n.d.) and lowest (though still high) in South Africa (15 years, country average = 13 years; “Average years of schooling”, n.d.) likely due to convenience sampling occurring at academic research institutions. WAMI Index scores were also higher than the general population (0.58, Psaki et al., 2014), highest in the United States (0.83) and Netherlands (0.82) and lowest in India (0.68), albeit still higher than the general population.

**Table 2.** Sociodemographic characteristics of healthy adult participants across sites

	Site					*F*/χ^2^	*p*	Post hoc^a^
	Brazil	India	Netherlands	South Africa^b^	United States			
*N*	53	50	50	52	51			
Mean age (SD)	31.96 (7.49)	27.70 (5.08)	32.70 (9.71)	29.79 (9.36)	27.82 (7.16)	4.27	0.002	Netherlands > India
Sex (% male)	20 (37.7%)	27(54.0%)	23 (46.0%)	18 (34.6%)	20 (39.2%)	5.00	0.29	
Mean WAMI (SES) (SD)	0.73 (0.09)	0.68 (0.11)	0.82 (0.07)	0.77 (0.09)	0.83 (0.06)	28.76	<0.001	United States = Netherlands > Brazil = India; Netherlands = South Africa; United States > South Africa > India
Mean education (SD)	17.11 (2.59)	15.92 (2.16)	15.86 (2.86)	14.79 (2.45)	16.55 (1.68)	6.96	<0.001	Brazil = United States > South Africa

Abbreviations: χ^2^, chi-square; *F*, ANOVA *F* statistic; SD, standard deviation; SES, socioeconomic status derived from WAMI.

a Sites shown with = indicate they were not significantly different at *p* < 0.05. Sites shown with > indicate significant difference at *p* < 0.05.

b One participant at South Africa did not have intelligence scores and was dropped from further analyses.

### Summary statistics for IQ measures across sites

The distribution of raw FSIQ scores at each site fell within the expected ranges (Supplementary Figure S1). Means of FSIQ, VIQ and PIQ (Table 3) were generally higher at every site than the standard population mean of 100 but had standard deviations ranging from 10.7 to 12.9 as expected. There were no differences between IQ indices between sites when controlling for site differences in demographics (Table 3, for raw data in Table 3: Supplement, Supplementary Table S2 and Supplementary Figure S1) (FSIQ: *p* = 0.46, *η^2^* = 0.016; VIQ: *p* = 0.54, *η^2^* = 0.012 and PIQ: *p* = 0.20, *η^2^* = 0.025).

**Table 3.** Mean intelligence scores across sites controlling for biological sex, years of education and SES

	Site					*F*	*p*
	Brazil	India	Netherlands	South Africa	United States		
Mean FSIQ (SD)	103.57 (13.07)	104.57 (16.70)	110.978 (13.73)	109.017 (12.36)	107.921 (17.49)	0.92	0.46
Mean VIQ (SD)*	104.50 (15.13)	–	112.10 (12.38)	114.69 (12.55)	109.66 (15.10)	3.38	0.72
Mean PIQ (SD)*	101.95 (18.02)	–	108.93 (14.87)	103.14 (15.05)	105.08 (18.19)	1.58	0.20

Abbreviations: *F,* ANOVA *F* statistic; FSIQ, full-scale intelligence quotient; PIQ, perceptual intelligence quotient; VIQ, verbal intelligence quotient.

*Note*: Unadjusted results in the Supplementary Table S2.

* The Binet Kamat Test does not provide index scores for verbal or perceptual reasoning.

### Convergent and discriminant validity

**Figure 1.**
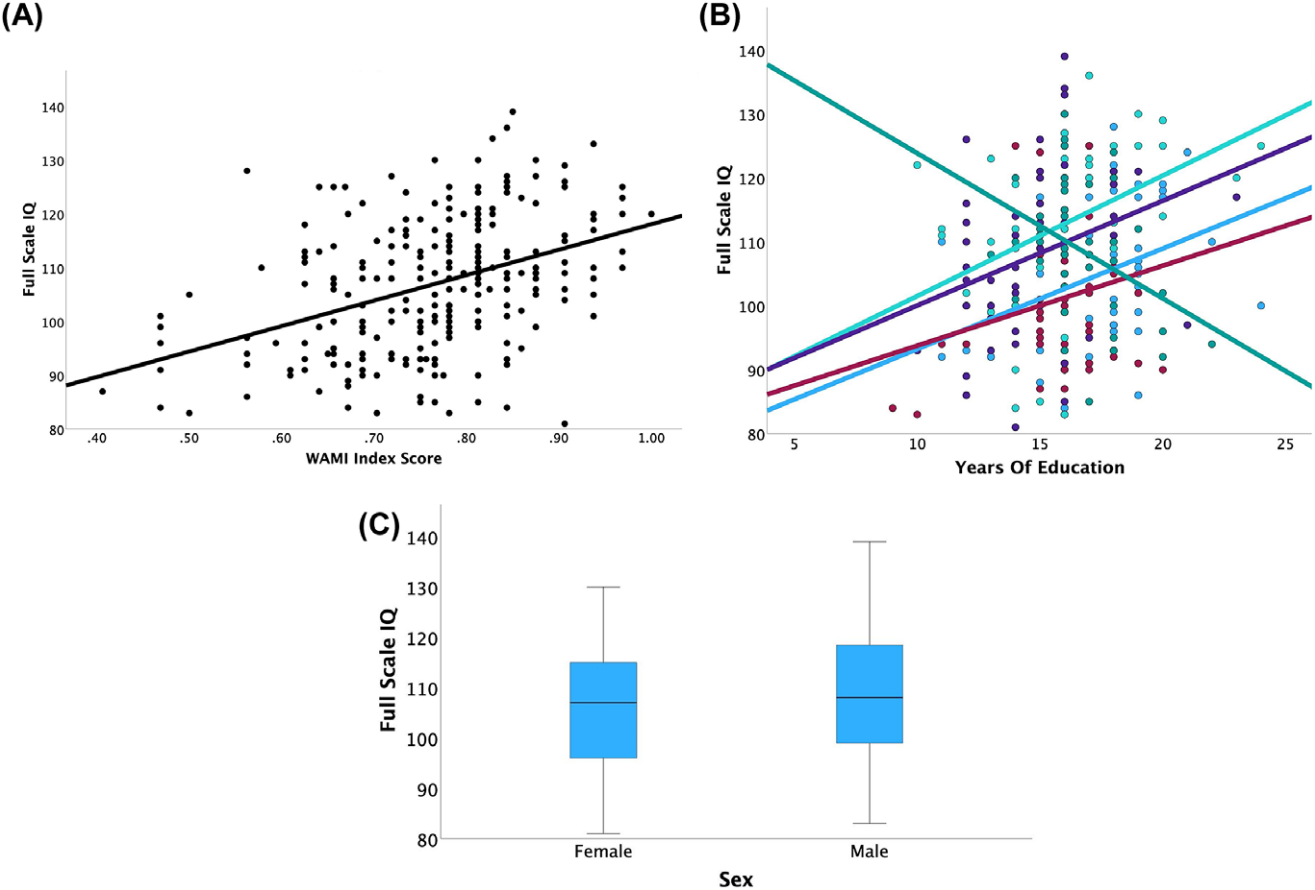
Associations between FSIQ scores and sociodemographics. (A) Main effect of WAMI index score (SES) was significant. (B) Full-scale IQ score is positively associated with educational attainment in Brazil (blue), India (maroon), Netherlands (teal) and South Africa (purple) and negatively associated with educational attainment in the United States (green). Main effects of (C) sex were not significant. Figures depicting individual sites in the Supplementary Figure S2. SES, socioeconomic status from the WAMI; FSIQ, full-scale IQ.

Consistent with the convergent validity hypotheses, we find higher SES, as measured by the WAMI index, was significantly associated with increased FSIQ scores (*F*(1,230) = 12.48, *p* < 0.001; partial *η^2^* = 0.051) and this was consistent by site as shown by the lack of interaction and respective small effect size of SES by site (*F*(4,222) = 0.61, *p* = 0.66, partial *η^2^* = 0.011). Specifically, FSIQ increased 0.32 points for every 1 standard deviation (i.e., 0.10 point) increase in WAMI index score (Figure 1A and Table 4).

**Table 4.** ANOVA predicting FSIQ scores

Source	SS	df	*F*	Partial η^2^	*p*-Value
*Covariate*					
Site	423.90	4	0.917	0.016	0.46
Age	24.52	1	0.212	0.001	0.65
Sex	343.42	1	2.972	0.013	0.09
Years of education	267.65	1	2.316	0.010	0.13
SES	1,442.20	1	12.481	0.051	**<0.001**
Age*site	485.77	4	1.051	0.018	0.38
Sex*site	380.53	4	0.823	0.014	0.51
Education*site	1,578.24	4	3.415	0.056	**0.01**
SES*site	282.03	4	0.610	0.011	0.66
Error	26,575	230			
Total	2,956,596	255			

Abbreviations: df, degrees of freedom; SES, socioeconomic status from the WAMI; SS, type III sum of squares.

Adjusted *R* ^2^ = 0.23.

The results of the convergent validity hypothesis for years of education were mixed because the effect of education on FSIQ was found to differ significantly by site (*F*(4,230) = 3.42, *p* = 0.01, partial *η^2^* = 0.056), such that each country showed a positive association between years of education and FSIQ, except the United States. Specifically, FSIQ increased 9.45 points in Brazil, 4.35 points in India, 10.73 points in the Netherlands and 4.52 points in South Africa for every standard deviation (2.5-year) increase in education; however, in the United States, FSIQ decreased by 13.52 points for every 2.5-year increase in education (Figure 1B).

Consistent with the discriminant validity hypotheses, we found that (*F*(1,230) = 0.21, *p* = 0.64, *η^2^* = 0.001) sex (*F*(1,230) = 2.97, *p* = 0.09, *η^2^* = 0.013) was not associated with FSIQ (Figure 1C). Also, results for convergent and discriminant validity hypotheses were consistent for both VIQ and PIQ (Supplementary Table S3 and Supplementary Figures S2 and S3). Finally, sensitivity analyses including additional control for a measure of language proficiency did not change this pattern of results and was itself not found to be a significant predictor of FSIQ, VIQ or PIQ (Supplementary Table S4). Finally, excluding data from the South Africa and India sites did not alter the findings (Supplementary Table S5).

## Discussion

Herein, we described the collection of harmonized IQ data for use in a large-scale, multisite, global study. Researchers conducting this study performed considerable prospective harmonization procedures prior to the onset of data collection to ensure the compatibility of IQ scores across sites. Prospective harmonization included consultation with local experts and attempts to utilize a single family of tests (i.e., Wechsler tests) in as many sites as possible, with the goal of yielding largely compatible measures with country-specific norms. Consistent with our discriminant validity hypotheses, associations between sex and IQ were not detected in this healthy participant sample. Consistent with our convergent validity hypothesis, higher FSIQ, as well as VIQ and PIQ, were associated with higher SES across the entire sample. The hypothesized positive association with education was confirmed in four of the five sites but did not hold for the United States. Validation of the prospectively harmonized IQ measure developed in this study provides preliminary support for using such an approach in future studies.

SES is known to be closely tied to socioenvironmental improvements, and correlates of lower SES such as lack of access to clean water/sanitation (Dearden et al., 2017; Orgill-Meyer and Pattanayak, 2020) fewer household assets or resources (Hackman et al., 2010; Barreto et al., 2017; Flensborg-Madsen et al., 2020; Zhang, 2021), and lower maternal educational attainment (Lawlor et al., 2006; Crookston et al., 2014; Lewinn et al., 2020) are closely tied to lower IQ scores in previous global cohort studies of children and youth. In our global study that collected data from healthy adult participants in five sites spanning five continents, IQ increased an average of three points for every 0.1-point increase in SES (WAMI index score) across these five study sites. This finding that higher SES was associated with higher IQ across sites adds to the knowledge base by showing convergently valid, stable associations between these factors across a broad range of SES indicators in a multinational context. Moreover, our finding that IQ scores did not associate with sex is also consistent with prior findings (Colom et al., 2002; Miller et al., 2009; Baxendale, 2011; Daseking et al., 2017; Halpern and Wai 2019; Pezzuti et al., 2020) and contributes to the confidence that the procedural harmonization across sites did not introduce any type of systematic bias related to participant characteristics. Given that sex (Weber et al., 2021) could introduce bias in global health studies, our finding that IQ was not associated with sex suggests that our data are robust to these potential demographic biases.

Prospective harmonization yielded strong data compatibility in IQ but is not a panacea. As seen in our results, other procedures still may be needed to control differences in IQ across sites. Specifically, we found that associations between education and IQ (FSIQ, VIQ) varied by site such that higher levels of education were positively associated with IQ at every site except the United States, indicating site-specific associations (Teasdale and Owen, 2005; Dutton et al., 2016; Bratsberg and Rogeberg, 2018; Acosta et al., 2019) between education and IQ measures that could not be controlled by prospective harmonization. There are several possible interpretations for our finding of a site by education interaction effect. First, these findings may suggest that increasing years of education in the United States (beyond 12 years of compulsory education, e.g., community college) may not be as associated with increasing IQ scores as they are in other countries. Alternatively, given the nature of our convenience sample, it is likely that our participants are not representative of the U.S. population. Supporting this, previous research has reported associations between educational attainment, IQ and SES in U.S. samples similar to those observed at our Brazil, Netherlands, South Africa and India sites (Ritchie and Tucker-Drob, 2018). As such, our finding of a site by education interaction effect on IQ scores warrants further investigation in larger samples of more diverse participants in the United States.

Our study also examined expected associations with discrete ability across sites. Convergent and discriminant validity hypotheses were confirmed for both VIQ and PIQ consistent with prior studies (Mascie-Taylor and Gibson, 1978; Reynolds et al., 1987; Shuttleworth-Edwards et al., 2004; Mani et al., 2013).

This study had particular strengths in its prospective harmonization process and large-scale, multinational research design. We were able to leverage measures of both personal educational attainment and a globally sensitive measure of SES to examine the effects of these variables across sites and on FSIQ, VIQ and PIQ. At the same time, the study also had limitations. First, this study consisted of primarily convenience sample, including participants who responded to advertisements and were willing to volunteer to contribute to research as healthy individuals. We acknowledge that this limits the generalizability of our findings to those individuals with both the means and ability to present to multiple study visits and participate in all aspects of a study. In a cross-national context, this becomes even more salient as some participants may be unintentionally excluded due to lack of adequate time or transportation or mistrust in research programs. Future examination of the validity of the IQ score harmonization would benefit from more participants enrolled from wider catchment areas with potentially more study sites within countries that are housed in rural areas or off university campuses. Second, VIQ and PIQ were not available for the India site due to differential IQ assessment procedures. Third, evaluators at different sites had different levels of experience and training in the provision of IQ assessments, which could have influenced our findings. However, influences of differential training were mitigated by rigorous data-checking procedures occurring both within and between study sites and over the course of the study. Our study was not able to account for nuanced differences in language proficiency that may have influenced performance on IQ measures. However, sensitivity analyses examining basic language proficiency in the test language were performed and did not influence our results. Future studies should examine the influence of language proficiency in more detail as it may be associated with cross-national harmonization. Also, our study did not include participants with FSIQ less than 80; and therefore, we cannot presume that our findings are generalizable to individuals across the lower end of the IQ spectrum. Future studies would benefit from inclusion of these individuals to better understand how sociodemographic variables do or do not associate with IQ among the intellectually challenged. Finally, local norms were used in Brazil and India whereas publisher norms were used in the United States, South Africa and the Netherlands. Further, publisher norms standardized in the United States were used in South Africa because no local norms were available. We acknowledge that the use of local norms can substantially influence intelligence scores when compared to using publisher norms with the same population (Duggan et al., 2019) and that local norms may not accurately reflect broader population demographics in the same way as publisher norms (Fernández and Abe, 2018). However, sensitivity analyses excluding the South Africa site were performed and did not influence our results. Future studies should examine if validity statistics change when using publisher norms (versus local norms) for the purposes of harmonization.

## Conclusions

This study examined a harmonized measure of intelligence for use in a large, multinational study. Both convergent and discriminant validity of the IQ score with demographic variables were demonstrated. Our study provides preliminary support that prospective harmonization methods are effective in addressing data compatibility across multinational sites. This validated prospective harmonization offers future studies a blueprint for developing harmonizable, culturally relevant assessment tools across global study sites.

## Supporting information

DeSerisy et al. supplementary material DeSerisy et al. supplementary material

## Data Availability

Data are available upon written request.

## Supplementary material

The supplementary material for this article can be found at https://doi.org/10.1017/gmh.2024.22.
