# Supplementary material for: Assessing harmonized intelligence measures in a multinational study
Source: Glob Ment Health (Camb). 2024 Feb 23;11:e22. doi: 10.1017/gmh.2024.22 (PMC10988151; doi:10.1017/gmh.2024.22)
Supplement: DeSerisy et al. supplementary material [file S2054425124000220sup001.docx]

**Supplementary Results**

**Between countries comparison of Demographics**

Participants in the Netherlands were significantly older than participants in India (*p*=0.05) and the United States (*p*=0.05) and had higher FSIQ than participants from Brazil (*p*=0.02) and India (*p*<0.001). South African participants had fewer years of education than those from Brazil (*p*<0.001) and those from the United States (*p*=0.008). Participants in the United States had significantly higher WAMI index scores than participants at every other site except the Netherlands (Brazil: *p<*0.001; India: *p<*0.001; Netherlands: *p*=0.93; South Africa: *p*=0.007)*;* whereas, Indian participants had significantly lower WAMI index scores than participants in all sites except Brazil (Netherlands: *p<*0.001; South Africa: *p<*0.001).

**Table S1. Ethnoracial Backgrounds of Included Participants**

|  | Site | | | | | |
| --- | --- | --- | --- | --- | --- | --- |
|  | | Brazil | India | Neth. | S. Africa | U.S. |
| Ethnicity | | % within site | % within site | % within site | % within site | % within site |
| Non-Hispanic | | 98.1 | 100 | 96.0 | 100 | 82.4 |
| Caribbean | | - | - | - | - | 3.9 |
| Central American | | - | - | - | - | 2.0 |
| South American | | - | - | - | - | 9.8 |
| Spanish | | - | - | - | - | 2.0 |
| Hispanic | | 1.9 | - | 4.0 | - | 17.6 |
| Race | |  |  |  |  |  |
| White | | 52.8 | - | 96.0 | 50.0 | 47.1 |
| East Asian | | 3.8 | - | - | - | 17.6 |
| South Asian | | - | 100 | - | - | 7.8 |
| Black | | 17.0 | - | - | 11.5 | 19.6 |
| Mixed/Other | | 26.4 | - | 4.0 | 38.5 | 7.8 |

*Note: Neth.=Netherlands, S. Africa=South Africa, U.S.=United States, %=percent*

**Table S2.** **Mean Raw Intelligence Scores Across Sites**

|  | Site | | | | | | F | p | Post Hoc^a^ |
| --- | --- | --- | --- | --- | --- | --- | --- | --- | --- |
|  | | Brazil | India | Neth. | S. Africa | U.S. |  |  |  |
| Mean FSIQ (SD) | | 104.42 (11.58) | 101.16 (10.69) | 112.56 (12.70) | 107.88 (12.90) | 108.98 (10.52) | 7.00 | <0.001 | Neth.>Brazil=India; US>India |
| Mean VIQ (SD)* | | 106.45 (11.04) | - | 113.44 (12.58) | 112.25 (13.13) | 109.92 (11.26) | 3.40 | 0.02 | Neth.>Brazil, |
| Mean PIQ (SD)* | | 101.21 (14.24) | - | 109.16 (14.89) | 101.38 (14.01) | 105.59 (11.50) | 3.92 | 0.01 | Neth.>Brazil=SA |

*Note. FSIQ = full scale intelligence quotient, Neth. = Netherlands, S. Africa = South Africa, PIQ = perceptual intelligence quotient, U.S. = United States, VIQ = verbal intelligence quotient, F*= ANOVA F Statistic

**The Binet Kamat test does not provide index scores for verbal or perceptual reasoning.*

1. *Sites shown with = indicate they were not significantly different at p<0.05. Sites shown with > indicate significant difference at p<0.05*

**Table S3. ANOVAs Predicting VIQ and PIQ Scores**

| *Model Predicting VIQ Scores** | | | | | |  |
| --- | --- | --- | --- | --- | --- | --- |
| Source | SS | df | F | partial η^2^ | p-value | |
| *Covariate* |  |  |  |  |  | |
| Site | 264.28 | 3 | .72 | .012 | .54 | |
| Age | 372.40 | 1 | 3.04 | .016 | .08 | |
| Sex | 512.29 | 1 | 4.18 | .022 | .04 | |
| Years of Ed. | 241.41 | 1 | 1.97 | .011 | .16 | |
| SES | 827.37 | 1 | 6.75 | .035 | .01 | |
| Age*Site | 563.70 | 3 | 1.53 | .024 | .21 | |
| Sex*Site | 176.20 | 3 | .48 | .008 | .70 | |
| Ed.*Site | 1496.81 | 3 | 4.07 | .062 | .008 | |
| SES*Site | 509.96 | 3 | 1.39 | .022 | .25 | |
| Error | 22666 | 185 |  |  |  | |
| Total | 2531973 | 205 |  |  |  | |
| *Model Predicting PIQ Scores*** | | | | | |  |
| Source | SS | df | F | partial η^2^ | p-value | |
| *Covariate* |  |  |  |  |  | |
| Site | 831.34 | 3 | 1.58 | .025 | .20 | |
| Age | 19.67 | 1 | .11 | .001 | .74 | |
| Sex | 264.90 | 1 | 1.51 | .008 | .22 | |
| Years of Ed. | 85.70 | 1 | .49 | .003 | .49 | |
| SES | 595.17 | 1 | 3.39 | .018 | .07 | |
| Age*Site | 575.96 | 3 | 1.10 | .017 | .35 | |
| Sex*Site | 512.31 | 3 | .97 | .015 | .41 | |
| Ed.*Site | 1025.92 | 3 | 1.95 | .030 | .12 | |
| SES*Site | 559.24 | 3 | 1.06 | .017 | .37 | |
| Error | 32612 | 186 |  |  |  | |
| Total | 2279809 | 206 |  |  |  | |

*Note: PIQ=Perceptual Intelligence Quotient, VIQ=Verbal Intelligence Quotient, Ed.=education, SES=socioeconomic status from the WAMI, SS=Type III sum of squares, df= degrees of freedom, Neth.=Netherlands, S. Africa = South Africa, U.S = United States*

**Adjusted R^2^=.18*

***Adjusted R^2^=.11*

**Table S4. Sensitivity Analyses: ANOVAs Predicting IQ Scores Including Language Proficiency**

| *Model Predicting FSIQ Scores** | | | | | |
| --- | --- | --- | --- | --- | --- |
| Source | SS | df | F | partial η^2^ | p-value |
| *Covariate* |  |  |  |  |  |
| Site | 448.05 | 4 | .97 | .017 | .43 |
| Lang. Proficiency | 73.13 | 1 | .63 | .003 | .43 |
| Age | 25.80 | 1 | .22 | .001 | .64 |
| Sex | 337.97 | 1 | 2.92 | .013 | .09 |
| Years of Ed. | 273.08 | 1 | 2.36 | .010 | .13 |
| SES | 1466.75 | 1 | 12.66 | .053 | <.001 |
| Age*Site | 504.88 | 4 | 1.09 | .019 | .36 |
| Sex*Site | 399.58 | 4 | .86 | .015 | .49 |
| Ed.*Site | 1532.71 | 4 | 3.31 | .055 | .01 |
| SES*Site | 239.69 | 4 | .52 | .009 | .72 |
| Error | 26425 | 228 |  |  |  |
| Total | 2946992 | 254 |  |  |  |
| *Model Predicting VIQ Scores*** | | | | | |
| Source | SS | df | F | partial η^2^ | p-value |
| *Covariate* |  |  |  |  |  |
| Site | 290.39 | 3 | 1.64 | .03 | .18 |
| Lang. Proficiency | 259.72 | 1 | .79 | .013 | .50 |
| Age | 406.86 | 1 | 2.12 | .011 | .15 |
| Sex | 462.72 | 1 | 3.33 | .018 | .07 |
| Years of Ed. | 249.70 | 1 | 3.78 | .020 | .05 |
| SES | 862.48 | 1 | 2.04 | .011 | .16 |
| Age*Site | 572.90 | 3 | 7.05 | .037 | .009 |
| Sex*Site | 220.92 | 3 | 1.56 | .025 | .20 |
| Ed.*Site | 1408.68 | 3 | .60 | .010 | .61 |
| SES*Site | 442.66 | 3 | 3.84 | .059 | .01 |
| Error | 22381 | 183 |  |  |  |
| Total | 2521364 | 204 |  |  |  |
| *Model Predicting PIQ Scores**** | | | | | |
| Source | SS | df | F | partial η^2^ | p-value |
| *Covariate* |  |  |  |  |  |
| Site | 831.48 | 3 | 1.57 | .025 | .20 |
| Lang. Proficiency | .35 | 1 | .002 | .000 | .97 |
| Age | 25.42 | 1 | .14 | .001 | .71 |
| Sex | 291.56 | 1 | 1.65 | .009 | .20 |
| Years of Ed. | 87.68 | 1 | .50 | .003 | .48 |
| SES | 599.15 | 1 | 3.40 | .018 | .07 |
| Age*Site | 586.26 | 3 | 1.11 | .018 | .35 |
| Sex*Site | 482.15 | 3 | .91 | .015 | .44 |
| Ed.*Site | 1023.71 | 3 | 1.94 | .031 | .13 |
| SES*Site | 551.84 | 3 | 1.04 | .017 | .38 |
| Error | 32451 | 184 |  |  |  |
| Total | 2271528 | 205 |  |  |  |

*Note: FSIQ =* Full-Scale Intelligence Quotient, *Lang*. = Language, *PIQ=Perceptual Intelligence Quotient, VIQ=Verbal Intelligence Quotient, Ed.=education, SES=socioeconomic status from the WAMI, SS=Type III sum of squares, df= degrees of freedom, Neth.=Netherlands, S. Africa = South Africa, U.S = United States*

**Adjusted R^2^=.23*

***Adjusted R^2^=.19*

***Adjusted R^2^=.10*

**Table S5. Sensitivity Analyses: ANOVAs Predicting IQ Scores Excluding Data From India and South Africa Sites**

| *Model Predicting FSIQ Scores** | | | | | |
| --- | --- | --- | --- | --- | --- |
| Source | SS | df | F | partial η^2^ | p-value |
| *Covariate* |  |  |  |  |  |
| Site | 18.85 | 1 | .08 | .001 | .92 |
| Age | .78 | 2 | .01 | .000 | .94 |
| Sex | 110.20 | 1 | .92 | .007 | .34 |
| Years of Ed. | 80.84 | 1 | .68 | .005 | .41 |
| SES | 385.41 | 1 | 3.23 | .023 | .08 |
| Age*Site | 75.18 | 1 | .32 | .005 | .73 |
| Sex*Site | 1.31 | 2 | .01 | .000 | .99 |
| Ed.*Site | 1550.82 | 2 | 6.50 | .085 | .002 |
| SES*Site | 44.42 | 2 | .19 | .003 | .83 |
| Error | 16592.67 | 2 |  |  |  |
| Total | 1837432.00 | 139 |  |  |  |
| *Model Predicting VIQ Scores*** | | | | | |
| Source | SS | df | F | partial η^2^ | p-value |
| *Covariate* |  |  |  |  |  |
| Site | 92.20 | 2 | .39 | .006 | .68 |
| Age | 54.59 | 1 | .46 | .003 | .50 |
| Sex | 168.57 | 1 | 1.41 | .010 | .24 |
| Years of Ed. | 88.49 | 1 | .74 | .005 | .39 |
| SES | 470.25 | 1 | 3.93 | .027 | .050 |
| Age*Site | 140.89 | 2 | .59 | .008 | .56 |
| Sex*Site | .61 | 2 | .003 | .000 | .99 |
| Ed.*Site | 1496.29 | 2 | 6.25 | .083 | .003 |
| SES*Site | 446.76 | 2 | 1.87 | .026 | .16 |
| Error | 16632.23 | 139 |  |  |  |
| Total | 1880694.00 | 154 |  |  |  |
| *Model Predicting PIQ Scores**** | | | | | |
| Source | SS | df | F | partial η^2^ | p-value |
| *Covariate* |  |  |  |  |  |
| Site | 297.57 | 2 | .83 | .012 | .44 |
| Age | 104.65 | 1 | .58 | .004 | .45 |
| Sex | 7.88 | 1 | .04 | .000 | .83 |
| Years of Ed. | 76.51 | 1 | .43 | .003 | .52 |
| SES | 112.49 | 1 | .63 | .004 | .43 |
| Age*Site | 423.92 | 2 | 1.18 | .017 | .31 |
| Sex*Site | .68 | 2 | .002 | .000 | .99 |
| Ed.*Site | 933.22 | 2 | 2.60 | .036 | .08 |
| SES*Site | 100.69 | 2 | .28 | .004 | .76 |
| Error | 24964.09 | 139 |  |  |  |
| Total | 1735305.00 | 154 |  |  |  |

*Note: FSIQ =* Full-Scale Intelligence Quotient, *PIQ=Perceptual Intelligence Quotient, VIQ=Verbal Intelligence Quotient, Ed.=education, SES=socioeconomic status from the WAMI, SS=Type III sum of squares, df= degrees of freedom, Neth.=Netherlands, S. Africa = South Africa, U.S = United States*

**Adjusted R^2^=.17*

***Adjusted R^2^=.16*

***Adjusted R^2^=.07*


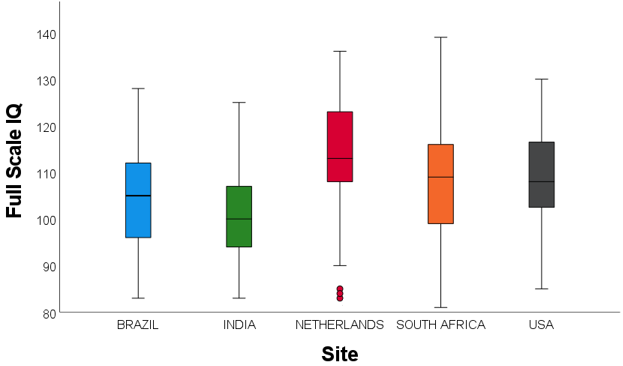


**Figure S1**. Unadjusted FSIQ across Sites. Participants in the Netherlands had higher FSIQ scores than participants in Brazil, India, and South Africa; participants in the United States also had higher FSIQ scores than participants in India.

*Note*: FSIQ=Full Scale Intelligence Quotient, USA=United States


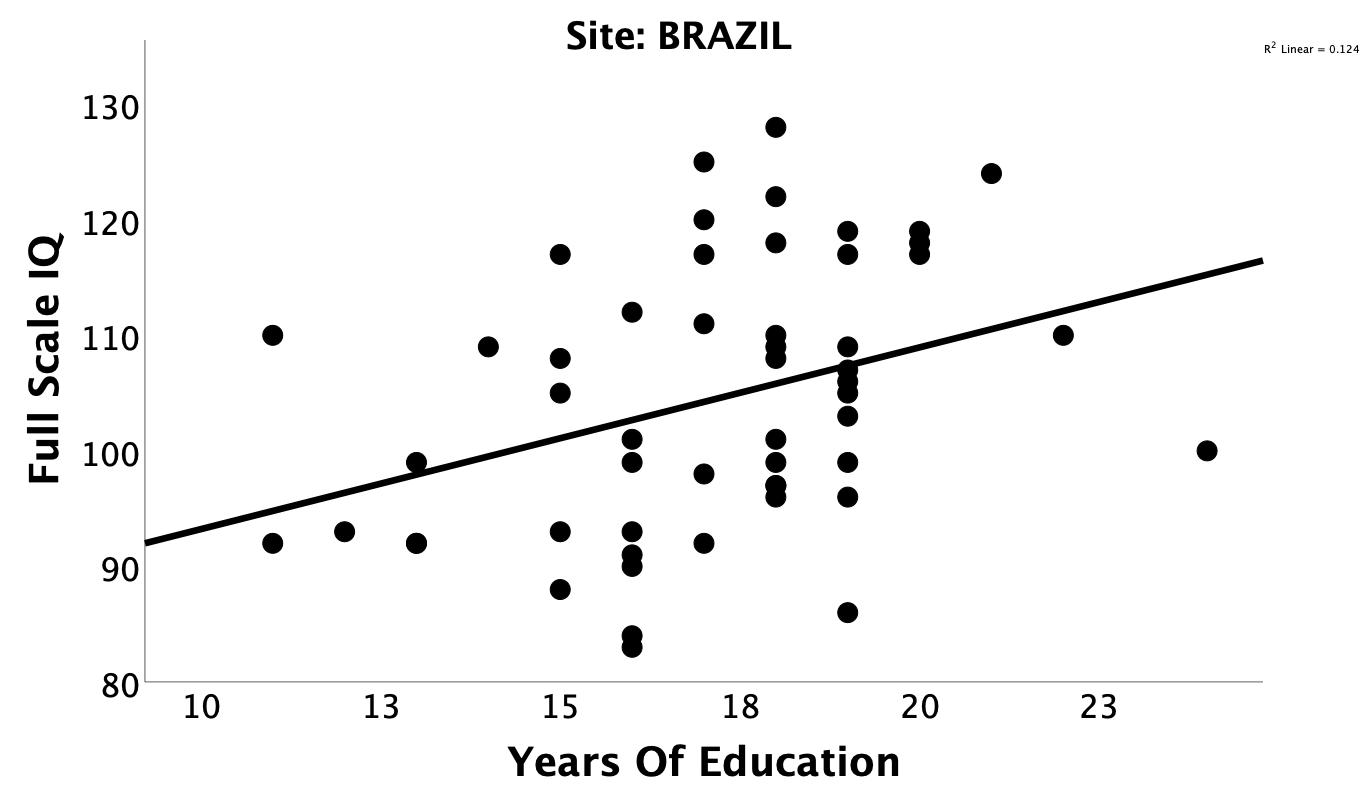


**(A)**


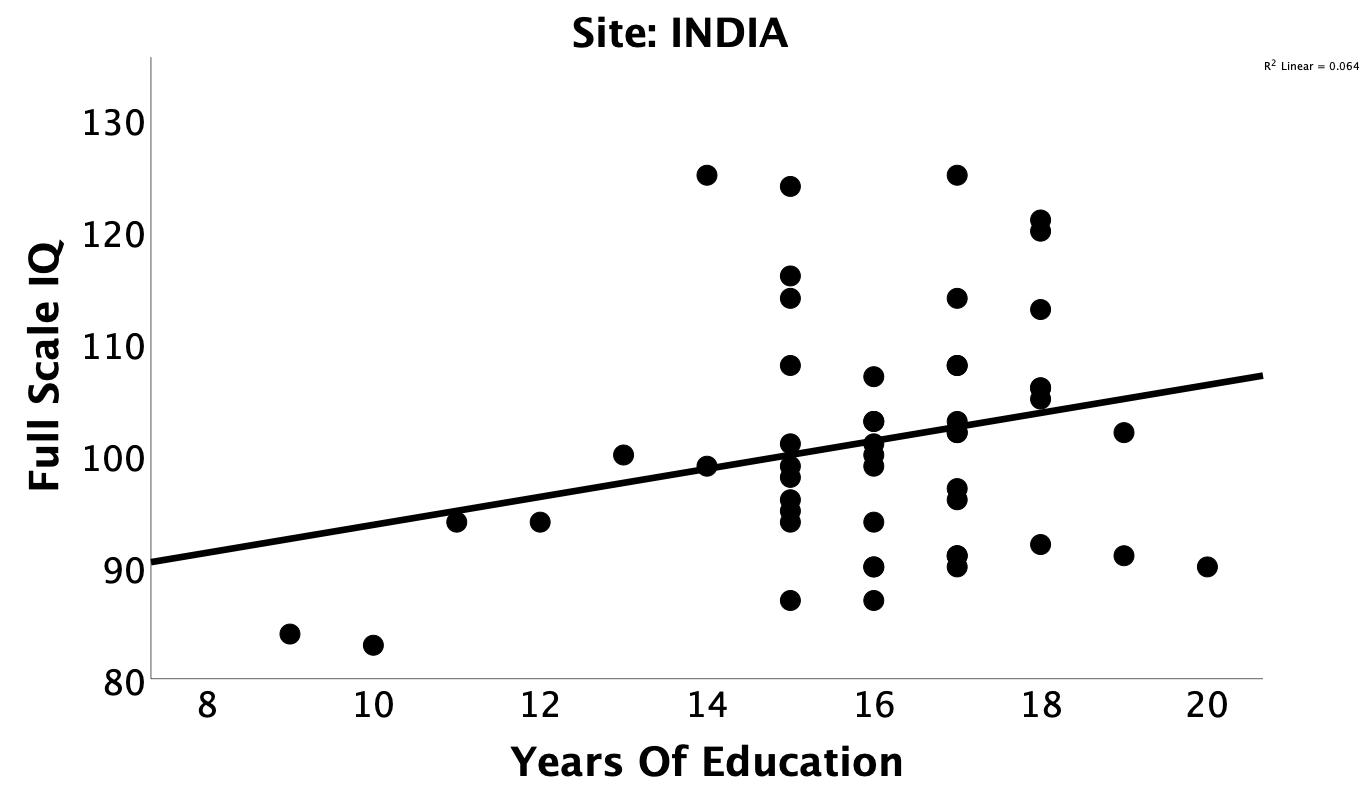


**(B)**


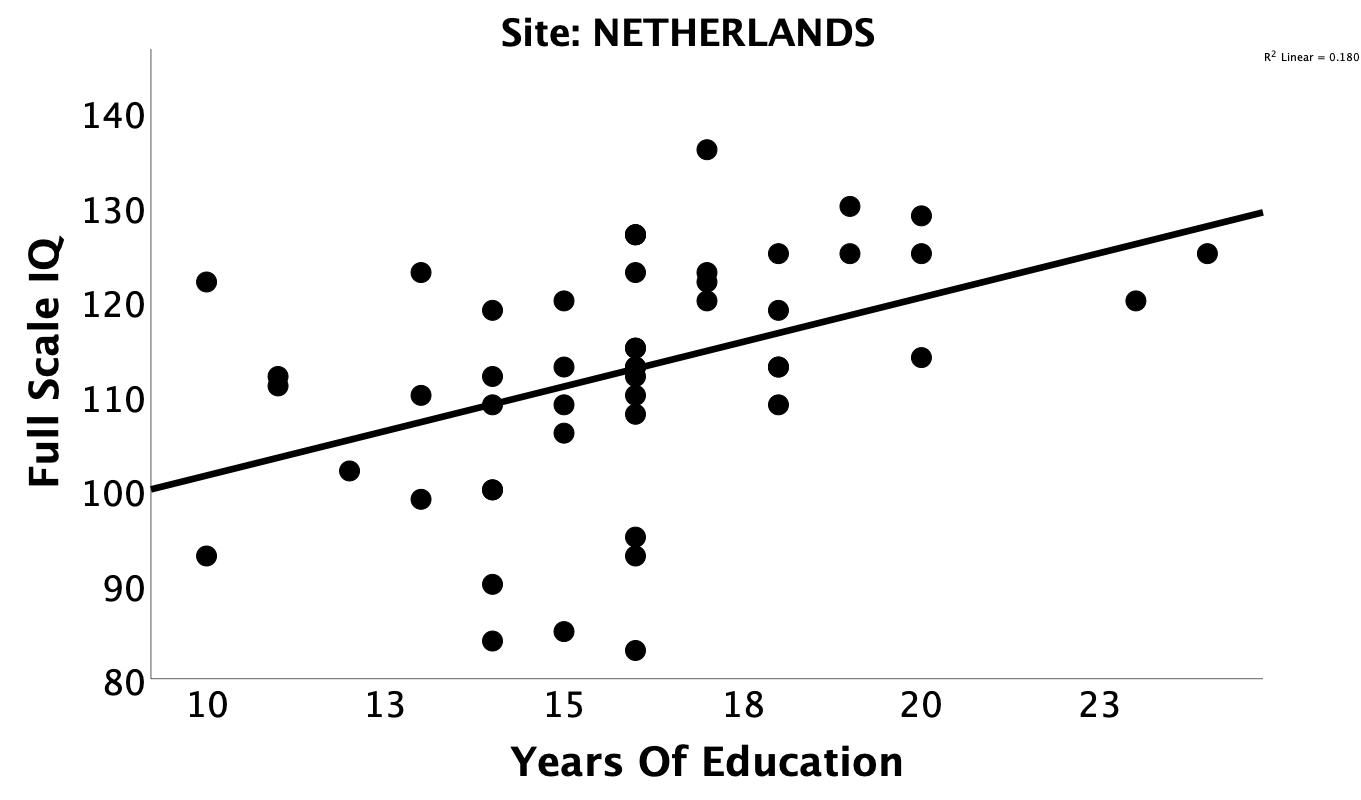


**(C)**


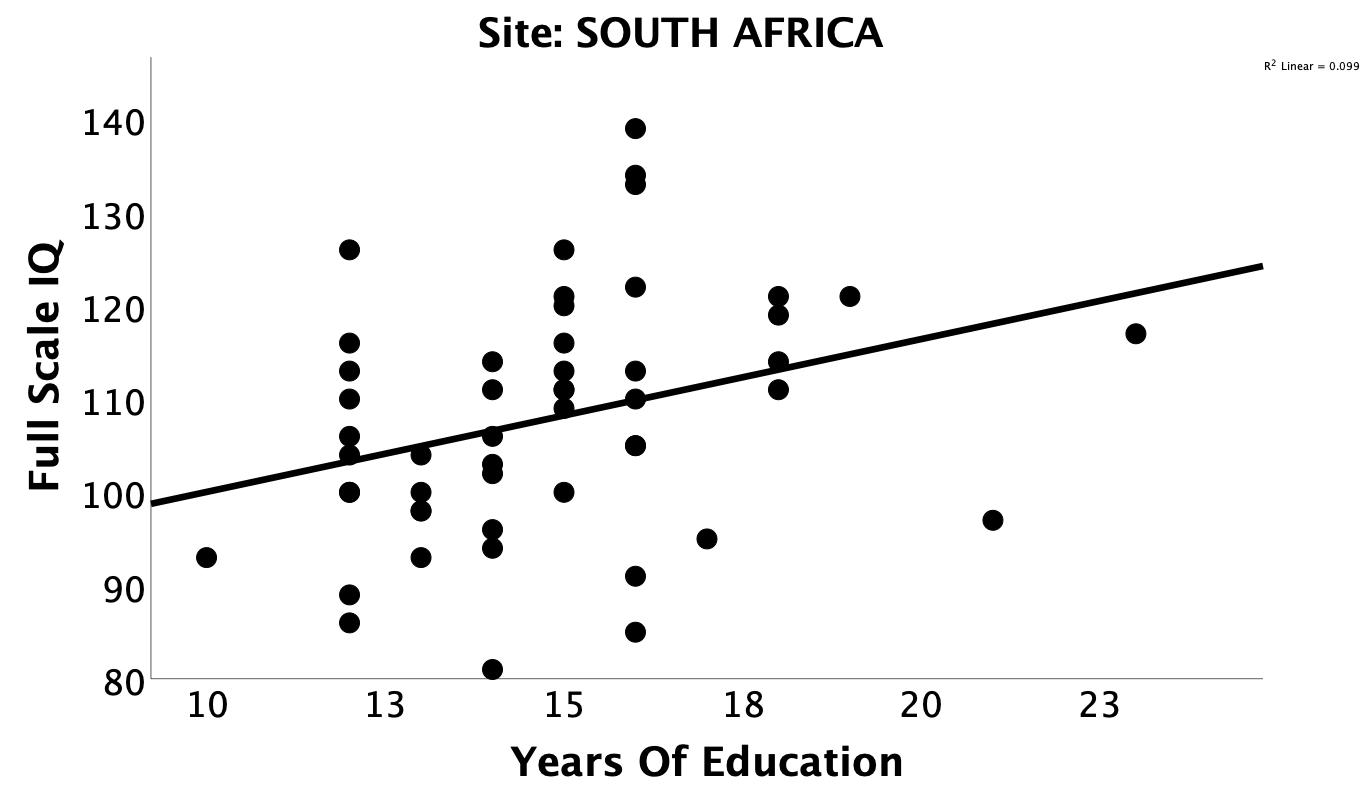


**(D)**

**Figure S2.** **Associations between FSIQ scores and Sociodemographics.** Full scale IQ score is positively associated with educational attainment in A) Brazil, B) India, C) Netherlands, and D) South Africa, and negatively associated with educational attainment in the E) United States.

*Note:* *FSIQ=Full Scale IQ.*


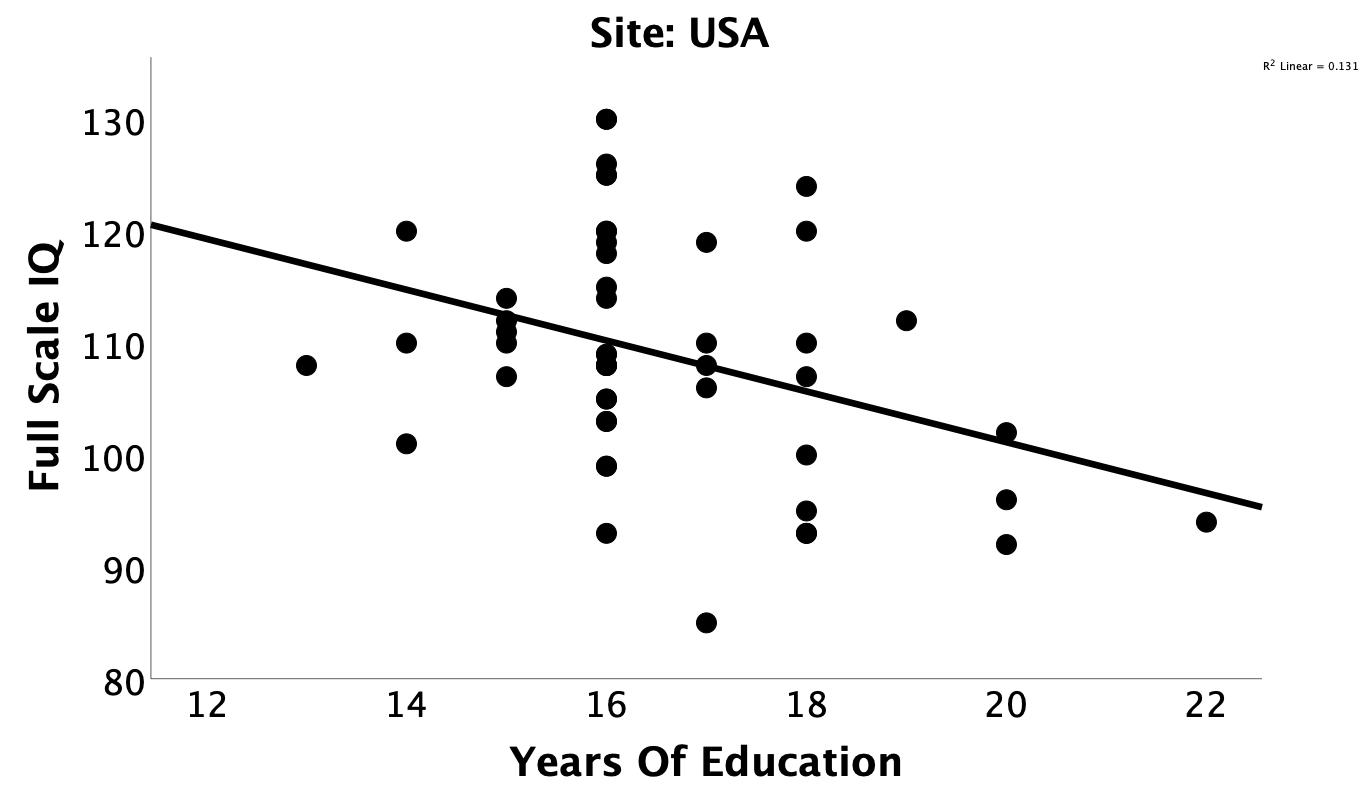


**(E)**

**
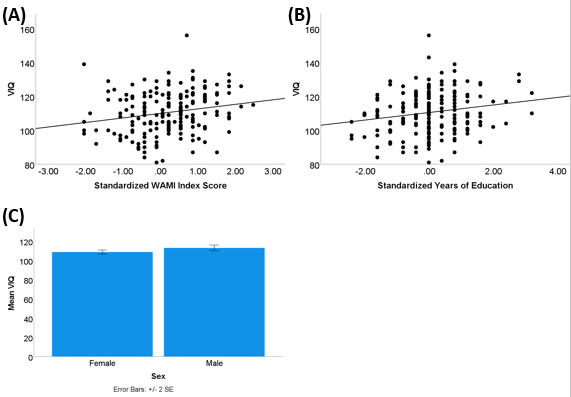
**

**Figure S3.** **Associations between VIQ scores and Sociodemographics**. A) Main effect of the WAMI index score (SES) was significant. Main effects of B) years of education, and C) sex were not significant.

*Note:* *SES=socioeconomic status from the WAMI, VIQ=Verbal Comprehension Index*


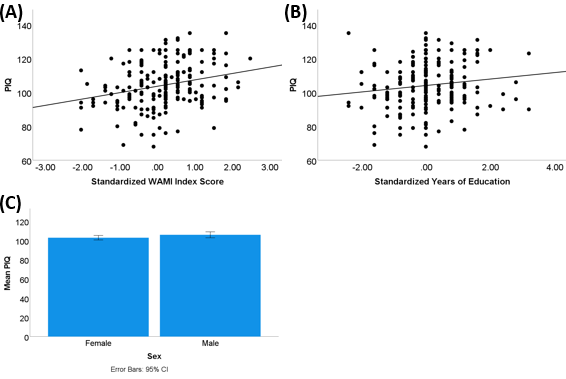


**Figure S4.** **Associations between PIQ scores and Sociodemographics**. A) Main effect of the WAMI index score (SES) was significant. Main effects of B) years of education, and C) sex were not significant.

*Note:* *SES=socioeconomic status from the WAMI, PIQ=Perceptual Reasoning Index*
