# Supplementary material for: Assessing harmonized intelligence measures in a multinational study
Source: Glob Ment Health (Camb). 2024 Feb 23;11:e22. doi: 10.1017/gmh.2024.22 (PMC10988151; doi:10.1017/gmh.2024.22)
Supplement: Peer review history

# Editor report: Recommendation: Assessing harmonized intelligence measures in a multinational study — R0/PR2

**DOI:** 10.1017/gmh.2024.22.pr2

**Affiliations:** Primary Healthcare Sciences, University of Zimbabwe Faculty of Medicine, Zimbabwe

No accompanying comment.

# Editor report: Decision: Assessing harmonized intelligence measures in a multinational study — R0/PR3

**DOI:** 10.1017/gmh.2024.22.pr3

**Affiliations:** Johns Hopkins University Bloomberg School of Public Health, United States

No accompanying comment.

# Editor report: Recommendation: Assessing harmonized intelligence measures in a multinational study — R1/PR5

**DOI:** 10.1017/gmh.2024.22.pr5

**Affiliations:** Primary Healthcare Sciences, University of Zimbabwe Faculty of Medicine, Zimbabwe

No accompanying comment.

# Editor report: Decision: Assessing harmonized intelligence measures in a multinational study — R1/PR6

**DOI:** 10.1017/gmh.2024.22.pr6

**Affiliations:** Johns Hopkins University Bloomberg School of Public Health, United States

No accompanying comment.
